# Increased Synaptic ATP Release and CD73-Mediated Formation of Extracellular Adenosine in the Control of Behavioral and Electrophysiological Modifications Caused by Chronic Stress

**DOI:** 10.1021/acschemneuro.2c00810

**Published:** 2023-03-07

**Authors:** Liliana Dias, Daniela Pochmann, Cristina Lemos, Henrique B. Silva, Joana I. Real, Francisco Q. Gonçalves, Daniel Rial, Nélio Gonçalves, Ana Patrícia Simões, Samira G. Ferreira, Paula Agostinho, Rodrigo A. Cunha, Angelo R. Tomé

**Affiliations:** † CNC—Center for Neuroscience and Cell Biology, University of Coimbra, 3004-504 Coimbra, Portugal; ‡ FMUC—Faculty of Medicine, University of Coimbra, 3004-504 Coimbra, Portugal; § Department of Life Sciences, Faculty of Sciences and Technology, University of Coimbra, 3004-517 Coimbra, Portugal

**Keywords:** ATP, adenosine, CD73, ecto-5′-nucleotidase, stress, hippocampus, prefrontal cortex, memory, mood, nerve terminals

## Abstract

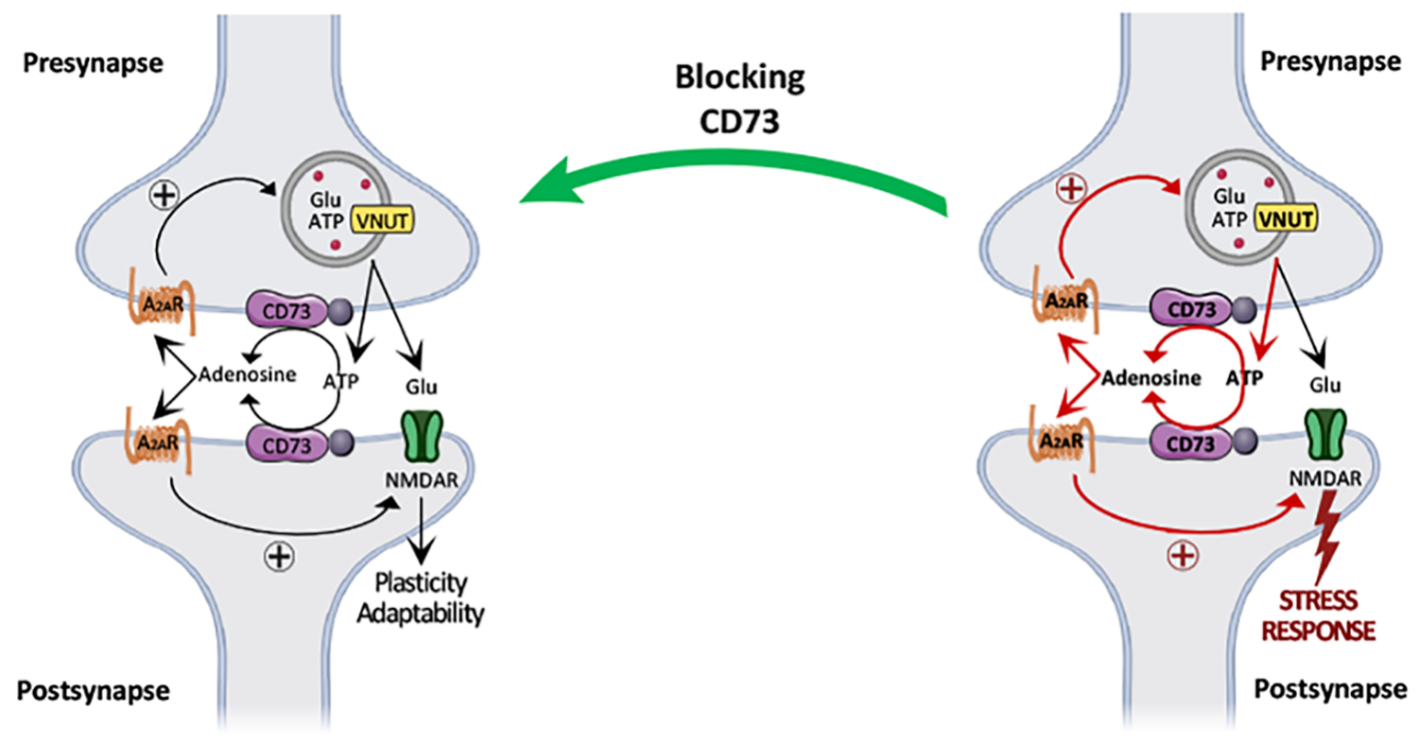

Increased ATP release and its extracellular catabolism through CD73 (ecto-5′-nucleotidase) lead to the overactivation of adenosine A_2A_ receptors (A_2A_R), which occurs in different brain disorders. A_2A_R blockade blunts mood and memory dysfunction caused by repeated stress, but it is unknown if increased ATP release coupled to CD73-mediated formation of extracellular adenosine is responsible for A_2A_R overactivation upon repeated stress. This was now investigated in adult rats subject to repeated stress for 14 consecutive days. Frontocortical and hippocampal synaptosomes from stressed rats displayed an increased release of ATP upon depolarization, coupled to an increased density of vesicular nucleotide transporters and of CD73. The continuous intracerebroventricular delivery of the CD73 inhibitor α,β-methylene ADP (AOPCP, 100 μM) during restraint stress attenuated mood and memory dysfunction. Slice electrophysiological recordings showed that restraint stress decreased long-term potentiation both in prefrontocortical layer II/III–layer V synapses and in hippocampal Schaffer fibers-CA1 pyramid synapses, which was prevented by AOPCP, an effect occluded by adenosine deaminase and by the A_2A_R antagonist SCH58261. These results indicate that increased synaptic ATP release coupled to CD73-mediated formation of extracellular adenosine contributes to mood and memory dysfunction triggered by repeated restraint stress. This prompts considering interventions decreasing ATP release and CD73 activity as novel strategies to mitigate the burden of repeated stress.

## Introduction

Purines are increasingly recognized as being involved in different physiopathological functions in the body,^1,2^ in particular as modulators fine-tuning information processing in neuronal networks.^3^ ATP is stored in synaptic vesicles and acts directly as a co-transmitter through P2 receptors^2,3^ and indirectly upon its extracellular conversion by ecto-nucleotidases into adenosine^4,5^ to activate adenosine receptors.^1,3^ Adenosine receptors in the brain are most abundantly located in synapses,^6−8^ namely, in excitatory glutamatergic synapses.^9^ Adenosine mainly acts through inhibitory adenosine A_1_ receptors and facilitatory adenosine A_2A_ receptors (A_2A_R)^10^ to assist encoding salience of information in neuronal circuits.^11^ This parallel activation of A_1_R and A_2A_R is ensured by different sources of adenosine: activation of A_1_R results from ATP/adenosine released from astrocytes,^12−17^ from microglia^18,19^ or from postsynaptic compartments of neurons through bidirectional nucleoside transporters,^20^ whereas synaptically released ATP-derived formation of extracellular adenosine through ecto-nucleotidases is selectively associated with the activation of adenosine A_2A_R controlling synaptic plasticity processes.^21−25^ Understanding the dynamics of the adenosine modulation system is relevant to control neurodegeneration^26^ in accordance with the view that chronic brain diseases begin by a dysfunction and damage of synapses (reviewed in refs (27) and (28)), mainly of excitatory synapses.^29,30^

The role of the purinergic system in conditions of mood dysfunction is still poorly characterized. On one hand, it has been established that the blockade of A_2A_R prevents moods dysfunction upon repeated stress,^31−33^ in accordance with the association of A_2A_R polymorphisms with the incidence of major depression.^34^ In parallel, there is robust evidence that P_2X7_R antagonism also prevents mood dysfunction upon repeated stress,^35−38^ also in accordance with the association of P_2X7_R polymorphisms with the incidence of major depression (reviewed in ref (39)). This implies an overfunction of purinergic A_2A_R and P_2X7_R, which apparently contrasts with the proposed antidepressant role of ATP based on the reported decreased ATP levels associated with depression.^40−43^ This is particularly surprising since ATP is a danger signal in the brain (reviewed in ref (44)), and there is an increased ATP release in different brain diseases,^22,23,45,46^ in particular in synapses that are particularly affected at the onset of depressive conditions (reviewed in ref (47)). Notably, the observed decrease of ATP release in depressive-like conditions was proposed to originate from astrocytes,^41,43,48,49^ which release ATP through lysosome exocytosis,^50^ through transmembrane Calhm2 channel proteins,^49^ through pannexin-1 channel,^48^ and through connexin-43,^51^ whereas the release of ATP from nerve terminals is mostly vesicular in nature.^52−54^ Furthermore, ATP release from astrocytes is designed to entrain a volume-like^55^ heterosynaptic depression^14,56^ and is expected to overshadow the vesicular release of ATP from nerve terminals, which only represent ∼1% of gray matter volume,^57^ designed to act within the synapse to bolster synaptic plasticity through A_2A_R activation after its local extracellular catabolism by ecto-nucleotidases.^21−25^

Thus, in view of the well-established role of synaptic A_2A_R overfunction to control aberrant plasticity associated with mood alterations, we now explored how the release of ATP from synapses was selectively affected in conditions of chronic stress and if an increased ATP-derived adenosine formation was also critical to sustain A_2A_R overfunction in excitatory synapses, as was previously observed in animal models of different brain diseases such as Alzheimer’s disease,^22^ Parkinson’s disease,^24,46^ epilepsy,^23^ fear memory,^25^ or fatigue.^58,59^

## Results and Discussion

### Increased Synaptic ATP Release upon Chronic Stress

**Figure 1:**
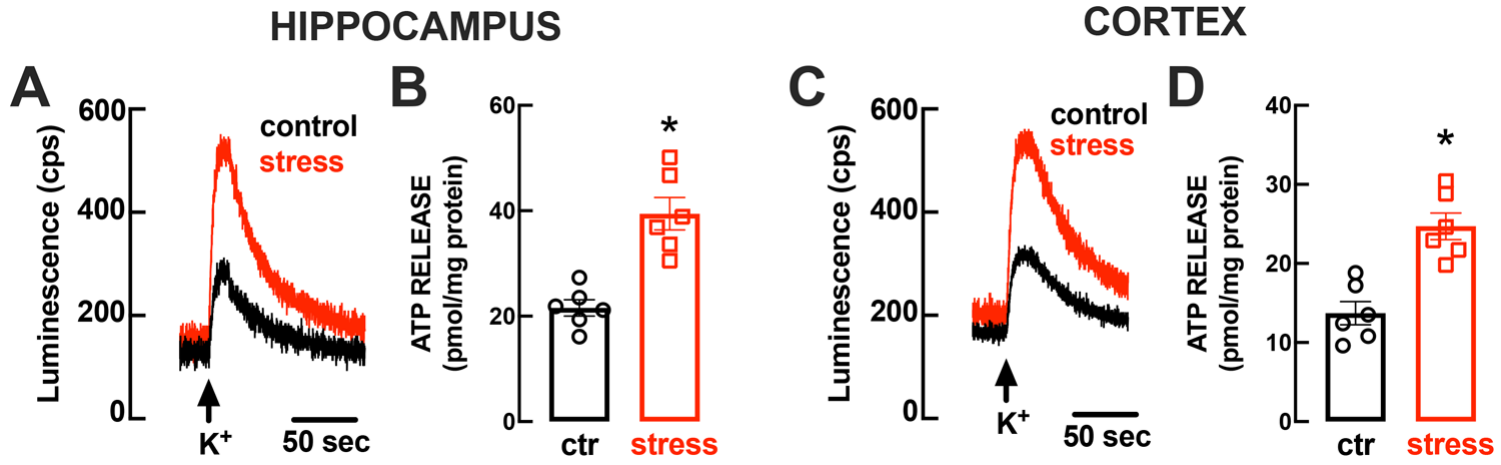
The evoked release of ATP from hippocampal and frontocortical nerve terminals is enhanced upon repeated stress. Male adult Wistar rats (8–10 weeks old) were subject to a protocol of restraint stress (4 h/day) during 14 days, evaluated behaviorally during 3 additional days, and then sacrificed for preparation of hippocampal and frontocortical synaptosomes. Representative recordings of luminescence emitted by luciferase as a measure of extracellular ATP in hippocampal synaptosomes (A) and in frontocortical synaptosomes (C) depolarized with addition of KCl (30 mM) in control (black) and in rats subjected to repeated restraint stress (red), which are quantified in (B) and (D) as the mean SEM of *n* = 6 different rats: *p < 0.05 two-tailed Student’s *t* test with Welsh correction vs control.

To selectively study the release of ATP from nerve terminals, we purified synaptosomes since the K^+^-induced release of ATP from synaptosomes reflects a vesicular release of ATP.^24,52,53^ We now report that the depolarization of rat hippocampal synaptosomes, by raising the extracellular concentration of K^+^ to 30 mM, triggered a rapid increase of extracellular ATP measured by its luminometric detection using the luciferin-luciferase assay (Figure 1A, black line). The average K^+^-evoked ATP release from hippocampal synaptosomes of control animals was 21.6 ± 1.6 pmol/mg protein (*n* = 6), a value similar to that reported previously by others using synaptosomes purified from different brain areas.^22,53^ As shown in the representative traces of Figure 1A, the K^+^-induced ATP release was larger in hippocampal synaptosomes collected from rats subject to repeated restraint stress, reaching a value of 39.5 ± 3.1 pmol/mg protein (*n* = 6, *t* = 5.173, *p* = 0.001 vs control). Likewise, the K^+^ (30 mM)-evoked release of ATP from frontocortical synaptosomes was also larger (*t* = 4.899, *p* < 0.001) in stressed rats (24.7 ± 1.7 pmol/mg protein, *n* = 6) compared to control rats (13.7 ± 1.5 pmol/mg protein, *n* = 6) (Figure 1C,D).

These results show that the evoked release of ATP in synapses is increased upon repeated stress, associated with mood and memory dysfunction (Figure 3), in clear opposition to the previously reported decreased release of ATP from astrocytes upon emergence of depressive-like behavior.^41,43,48,49^ These apparently contradictory findings should be interpreted in view of the different roles of ATP within synapses and between synapses: within synapses released ATP from nerve terminals sustains the ecto-nucleotidase-mediated formation of extracellular adenosine to selectively activate adenosine A_2A_R and bolster synaptic plasticity processes.^21−25^ In contrast, ATP released from astrocytes is mostly engaged in heterosynaptic depression mainly through the activation of A_1_R^12−17^ as well in the coordination of microglia recruitment through P_2Y12_R^60^ and microglia activation through P_2X7_R.^61^ Thus, astrocytically released ATP and synaptically released ATP fulfill parallel, different, and complementary roles in the global control of neuronal circuits and neuroinflammation and in the local control of synaptic plasticity, respectively. Importantly, it is unlikely that the two pools of ATP cross-contaminate each other’s function: in fact, the amount of ATP released from synapses is expected to be near negligible compared to the bulk of the tissue since synapses only represent 1–2% of the total gray matter volume,^57^ whereas astrocytically released ATP is unlikely to reach the synapse as such in view of the rapid and efficient activity of ecto-nucleotidases.^62,63^ In fact, we have previously shown that exogenously added ATP (as well as purportedly “stable” ATP analogues) does not reach synapses as ATP but rather as adenosine.^63^ Thus, the P_2_R-mediated antidepressant effect of exogenously added ATP^40−43^ is unlikely to be exerted directly within synapses but instead likely results from extra-synaptic actions, eventually associated with overactivation of A_1_R.^64^ This is further supported by our previous observations that although different P_2_Rs are located in hippocampal^65^ and striatal synapses,^24^ P_2_R antagonists have a limited impact on synaptic plasticity in the hippocampus^66^ or in the striatum^24^ hinting at a more relevant role of indirect and extra-synaptic rather than synaptic P_2_R to control information processing in cortical circuits.^67−69^

### Increased Synaptic Density of vNUT and CD73 upon Chronic Stress

**Figure 2:**
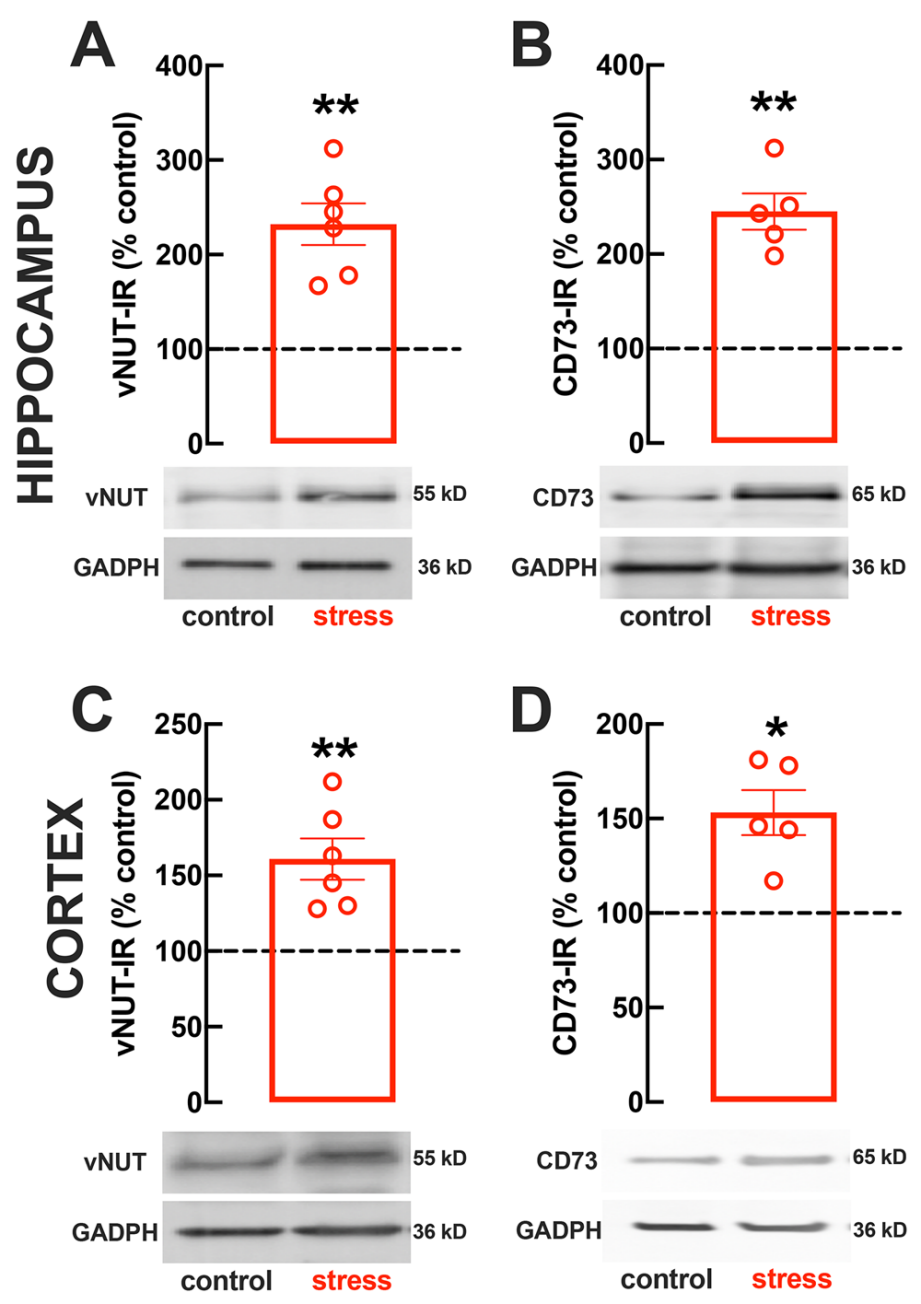
Repeated restrain stress triggered an increased density of vesicular nucleotide transporters and of ecto-5′-nucleotidase (CD73) in hippocampal and frontocortical nerve terminals. Male adult Wistar rats (8–10 weeks old) were subject to a protocol of restraint stress (4 h/day) during 14 days, evaluated behaviorally during 3 additional days, and then sacrificed for preparation of hippocampal and frontocortical synaptosomes. The immunoreactivity of vesicular nucleotide transporters (vNUT) (A, C) and of CD73 (B, D) was increased in synaptosomes from the hippocampus (A, B) and from the frontal cerebral cortex (C, D) of rats subject to repeated restraint stress (red) compared to control (black, equivalent to the dashed line corresponding to 100%), with representative Western blots shown below each bar graph. Data are the mean SEM of *n* = 6 different rats: *p < 0.05 and **p < 0.01 one-tailed Student’s *t* test vs 100% (control).

Since the loading of ATP into synaptic vesicles strictly requires the activity of vesicular nucleotide transporters (vNUT),^70^ we investigated if the increased ATP release upon repeated stress was paralleled by an increased density of vNUT. We observed that the density of vesicular nucleotide transporters vNUT in hippocampal synaptosomes of repeatedly stressed was 232 ± 22% larger than that of control rats (*n* = 6, *t* = 5.973, *p* = 0.002) (Figure 2A). Likewise, the density of vNUT in frontocortical synaptosomes of repeatedly stressed was 161 ± 14% larger than that of control rats (*n* = 6, *t* = 4.458, *p* = 0.007) (Figure 2C). This increased density of vNUT suggests that the observed increase of the evoked release of ATP from nerve terminals upon repeated stress might mostly result from a larger ready-releasable pool or from increased ATP loading into synaptic vesicles rather than from a higher efficiency of vesicular release, which might be preserved in view of the similar input–output curves observed in recordings of synaptic transmission in both prefrontocortical and hippocampal slices between control and repeatedly stressed rats (Figure 4A,D).

As shown in Figure 2A,B, the repeated stress-induced increase of ATP release from nerve terminals was also accompanied by an increased density of ecto-5′-nucleotidase (or CD73), both in hippocampal synaptosomes (245 ± 19% larger than in control, *n* = 5, *t* = 7.582, *p* = 0.002) and in frontocortical synaptosomes (153 ± 12% larger than in control, *n* = 5, *t* = 4.469, *p* = 0.011). These findings join previous observations that the synaptic density of CD73 is increased in other noxious conditions such as epilepsy,^23,71−74^ Alzheimer’s disease,^22^ Parkinson’s disease,^24,46^ or aging,^75^ reinforcing the concept that increased CD73 density is an adaptive feature of plastic synapses.^74,76,77^

### CD73 Blockade Prevents Stress-Induced Behavioral Modifications

We next tested if the increase of ATP release and of CD73 density in hippocampal and frontocortical synapses was feeding the A_2A_R overfunction that we previously documented to be associated with mood and memory impairment triggered by repeated stress.^32,78^ For this purpose, we tested if a prolonged treatment with the CD73 inhibitor αβ-methylene ADP (AOPCP) prevented the behavioral alterations caused by repeated restraint stress.

**Figure 3:**
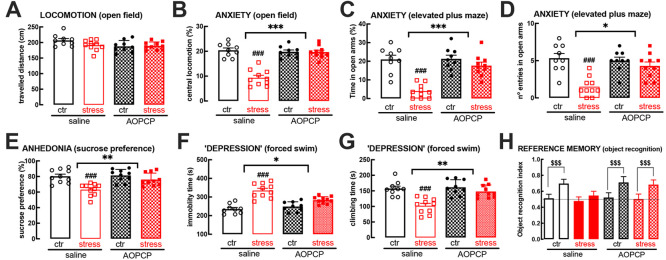
Male adult Wistar rats (8–10 weeks old) subject to a protocol of restraint stress (4 h/day) during 14 days displayed the expected features of depressed rats, which were prevented by the CD73 inhibitor α,β-methylene ADP (AOPCP). Compared to nonstressed rats (ctr-saline, open black bars), stressed rats displayed several behavioral modifications of mood and memory (stress-saline, open red bars), and whereas AOPCP intracerebroventricular continuous administration (100 μM, beginning 3 days before the stress protocol and until the sacrifice of the animals) was devoid of effects in nonstressed control rats (ctr-AOPCP, black dashed bars), AOPCP prevented all behavioral modifications of stressed rats (stress-AOPCP, red dashed bars): without modification of locomotor activity as evaluated in the open field (A), AOPCP prevented anxiety-like behavior as evaluated in the open field (B) and in the elevated-plus maze (C, D) tests, anhedonia as evaluated in the sucrose preference test (E), helpless-like behavior as evaluated by the forced-swimming test (F, G), and impaired memory performance as evaluated by the object-displacement test (H). Data are shown as the mean ± SEM; *n* = 9–10 rats per group: ^###^*P* < 0.001 effect of stress, *P < 0.05, **P < 0.01, and ***P < 0.001 effect of AOPCP using a Newman–Keuls multiple comparisons post hoc test after a two-way ANOVA; ^$$$^*P* < 0.001 comparing training (left bar of each pair) and test phase (right bar in each pair) in the object-displacement test.

The model of repeated restraint stress triggers robust and reproducible behavioral alterations of mood and memory in adult rats (Figure 3), and the continuous intracerebral infusion of the CD73 inhibitor αβ-methylene ADP (AOPCP, 100 μM, starting 3 days before the restraint stress protocol and present until sacrifice) was devoid of evident behavioral effects in control rats but attenuated or prevented the behavioral alterations caused by repeated stress (Figure 3). Thus, whereas there was no significant change of spontaneous locomotion (*t* = 1.917, *p* = 0.073, unpaired Student’s *t* test; Figure 3A), stressed rats displayed a thigmotaxic behavior indicative of an increased anxiety-like profile, as indicated by the decreased number of crossings in the central area of the open field, which was prevented by AOPCP (effect of stress *F*_1,34_ = 42.00, *p* < 0.001; effect of AOPCP *F*_1,34_ = 31.90, *p* < 0.001; interaction *F*_1,34_ = 40.37, *p* < 0.001; two-way ANOVA; Figure 3B). The stress-induced anxiety-like behavior was confirmed in the elevated plus maze where stressed rats displayed a decreased time in the open arms and a decreased number of entries in the open arms; AOPCP treatment attenuated the stress-induced decrease of the number of entries in the open arms of the elevated plus maze (effect of stress *F*_1,34_ = 31.98, *p* < 0.001; effect of AOPCP *F*_1,34_ = 14.68, *p* < 0.001; interaction *F*_1,34_ = 13.49, *p* < 0.001; two-way ANOVA; Figure 3C) and the stress-induced decrease of the time spent in the open arms of the elevated plus maze (effect of stress *F*_1,34_ = 20.03, *p* < 0.001; effect of AOPCP *F*_1,34_ = 5.933, *p* = 0.020; interaction *F*_1,34_ = 9.569, *p* = 0.004; two-way ANOVA; Figure 3D). Stressed rats also displayed an anhedonic behavior in the sucrose preference test, which was attenuated by AOPCP (effect of stress *F*_1,34_ = 19.09, *p* = 0.006; effect of AOPCP *F*_1,34_ = 7.459, *p* = 0.001; interaction *F*_1,34_ = 13.02, *p* = 0.025; two-way ANOVA; Figure 3E). The stress-induced increase of immobility in the forced swimming test, indicative of a depressive-like behavior, was also attenuated by AOPCP treatment (effect of stress *F*_1,34_ = 53.96, *p* < 0.001; effect of AOPCP *F*_1,34_ = 4.340, *p* = 0.044; interaction *F*_1,34_ = 12.85, *p* = 0.001; two-way ANOVA; Figure 3F), as was the stress-induced decrease of the time climbing the wall in the forced swimming test (effect of stress *F*_1,34_ = 20.85, *p* < 0.001; effect of AOPCP *F*_1,34_ = 10.85, *p* = 0.002; interaction *F*_1,34_ = 7.784, *p* = 0.009; two-way ANOVA; Figure 3G). Finally, stressed rats also displayed a deteriorated short-term reference memory and AOPCP attenuated the stress-induced decrease of the relative time exploring a displaced object (*t* = 1.093, *p* = 0.095 between displaced and nondisplaced object in stressed rats treated with vehicle and *t* = 6.704, *p* < 0.001 between displaced and nondisplaced object in stressed rats treated with AOPCP, unpaired Student’s *t* test; Figure 3H).

These results show that CD73 inhibition with AOPCP was sufficient to prevent the mood and memory dysfunction caused by repeated restraint stress. Since we had previously shown that the overfunction of A_2A_R was critical for the expression of mood and memory dysfunction triggered by repeated stress^32,78^ and the activation of central A_2A_R is selectively ensured by the particular pool of extracellular adenosine derived from CD73 activity,^21,23,25,79^ the present findings suggest that the ATP-derived formation of extracellular adenosine might be critical to sustain A_2A_R overfunction responsible for mood and memory dysfunction upon repeated stress. The present contention in the context of repeated stress joins previous similar conclusions in the context of Alzheimer’s disease,^22^ Parkinson’s disease,^24,46^ epilepsy,^23^ fear memory,^25^ or fatigue,^59^ to prompt the overall conclusion that increased ATP release and increased CD73-mediated activity leading to overfunction of A_2A_R are a common maladaptive feature of the stressed brain contributing to the emergence of abnormal brain function in disease conditions. Such a new concept paves the way to consider manipulations of the vesicular release of ATP and of the activity of CD73 as new candidate strategies to manage brain dysfunction.

### CD73 Blockade Prevents Stress-Induced Alterations of Synaptic Plasticity

Synaptic dysfunction is at the core of initial changes in depressive conditions,^47^ as typified by decreased synaptic plasticity after repeated stress in the hippocampus^32,80^ and prefrontal cortex.^81,82^

**Figure 4:**
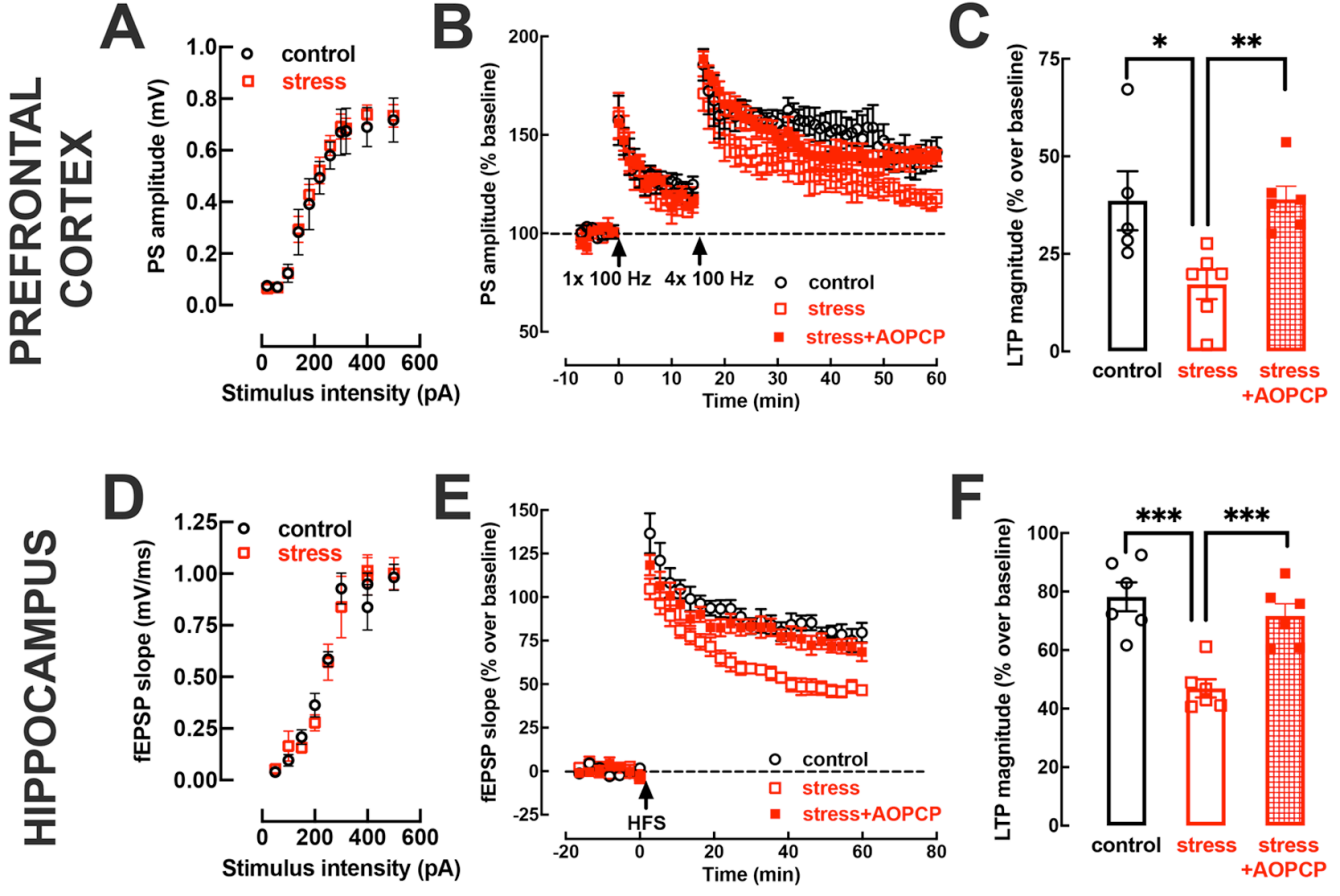
The impairment of synaptic plasticity by repeated restraint stress is prevented by the inhibition of CD73. Male adult Wistar rats (8–10 weeks old) were subject to a protocol of restraint stress (4 h/day) during 14 days, evaluated behaviorally during 4 additional days, and then sacrificed for preparation of prefrontocortical and hippocampal slices to electrophysiologically record synaptic transmission as population spike response (PS) between layer II/III and layer V (upper panels) and field excitatory potentials (fEPSP) between Schaffer fibers and CA1 pyramids (lower panels), respectively. The input/output curves were nearly superimposable between control (black symbols) and stressed rats (red symbols) in both prefrontocortical (A) and hippocampal slices (D), indicating a similar density of excitatory innervation in both brain areas in the two groups. However, upon application of a high-frequency stimulation (HFS, 5 trains of pulses of 3 s duration at 100 Hz in the prefrontal cortex and 100 Hz for 1 s in the hippocampus), stressed rats displayed a lower sustained increase of PS in the prefrontal cortex (B) or fEPSP in the hippocampus (E). This lower LTP magnitude in stressed rats was reverted to control values by acutely applying 100 μM AOPCP both in the prefrontal cortex (B, C) and in the hippocampus (E, F). Data are shown as the mean ± SEM; *n* = 5–6 rats per group: *P < 0.05, **P < 0.01, and ***P < 0.001, two-tailed Student’s *t* test with Welsh correction between indicated bars.

Accordingly, brain slices containing the prefrontal cortex, collected from rats subject to 14 days of repeated restraint stress, displayed a significantly (*t* = 2.532, *p* = 0.045) impaired synaptic plasticity, with an LTP magnitude (17.18 ± 3.76% over baseline, *n* = 6) lower than that recorded in the prefrontal cortex of control rats (38.62 ± 7.58% over baseline, *n* = 5) (Figure 4B,C), without alterations of the input–output curve (Figure 4A). Likewise, repeated stress also decreased LTP magnitude in the dorsal hippocampus (78.20 ± 4.92% in control and 46.92 ± 3.13% in stressed rats; *t* = 5.361, *p* = 0.006, *n* = 6) (Figure 4E,F) without altering the input–output curve (Figure 4D).

Notably, the superfusion of prefrontocortical and hippocampal slices with AOPCP (100 μM) reverted the decreased LTP magnitude triggered by repeated stress: thus, AOPCP recovered LTP magnitude both in prefrontocortical slices (17.18 ± 3.76% without and 38.97 ± 3.35% with AOPCP; *t* = 4.324, *p* < 0.002, *n* = 6) (Figure 4B,C) and in hippocampal slices (46.92 ± 3.13% without and 71.64 ± 4.19% with AOPCP; *t* = 4.730, *p* = 0.001, *n* = 6) from stressed rats (Figure 4E,F). The impact of blocking CD73 activity has mostly been associated with the elimination of the formation of ATP-derived extracellular adenosine, but guanine nucleotides may also be involved in the effects of AOPCP in the stressed brain given that stressful conditions can trigger the release of guanine nucleotides^83,84^ that can also be converted by CD73 into guanosine^85^ to exert an antidepressant-like action^86^ and modify neuronal function also through A_2A_R.^87^ To directly probe if the effect of AOPCP specifically involved extracellular adenosine, we tested the effect of AOPCP in the presence of adenosine deaminase to selectively remove endogenous extracellular adenosine. We observed that, whereas AOPCP recovered LTP magnitude in hippocampal slices of stressed rats (Figure 4E,F), AOPCP was devoid of effects on LTP magnitude in the presence of 2 U/mL adenosine deaminase (69.99 ± 3.97% without and 66.62 ± 4.95% with AOPCP; *t* = 0.7221, *p* = 0.497, *n* = 4), thus arguing for a critical involvement of extracellular adenosine in the effect of AOPCP. Additionally, the effect of AOPCP on LTP magnitude was phenocopied by the effect of A_2A_R blockade on LTP magnitude. Thus, in hippocampal slices from stressed rats, the A_2A_R antagonist SCH58261 (50 nM) also recovered LTP magnitude (46.07 ± 4.81% without and 70.23 ± 3.22% with SCH58261; *t* = 5.344, *p* = 0.006, *n* = 5). To directly assess if CD73 activity was associated with A_2A_R overfunction, we tested the impact of AOPCP upon blockade of A_2A_R blockade by SCH58261. We observed no additional effect of AOPCP on LTP magnitude when A_2A_R were blocked in hippocampal slices from stressed rats (70.23 ± 3.22% with SCH58261 and 73.48 ± 4.39% with SCH58261 and AOPCP; *t* = 0.5971, *p* = 0.567, *n* = 5).

These findings indicate that ATP-derived extracellular adenosine acting through A_2A_R is responsible for the stress-induced deterioration of hippocampal synaptic plasticity. This conclusion that AOPCP restores synaptic plasticity upon brain dysfunction triggered by repeated stress is aligned with previous observations that the inhibition of CD73 also restores synaptic plasticity in different brain areas under other conditions of brain dysfunction such as Alzheimer’s disease,^22^ Parkinson’s disease,^24,46^ or epilepsy.^23^ The parallel ability of AOPCP to correct behavioral dysfunction and cortical synaptic plasticity argues for the hypothesis that the dysfunction of synaptic plasticity might be a mechanistic basis of behavior dysfunction upon brain diseases, in accordance with the proposal that synaptic dysfunction might be a core process at the onset of brain diseases;^28^ this has been documented for Alzheimer’s disease,^27^ Parkinson’s disease,^88^ Huntington’s disease,^89^ Machado–Joseph’s disease,^90^ epilepsy^91^ as well as in major depression.^47^ Notably, A_2A_R blockade prevents synaptic and behavioral dysfunction in animal models of these brain diseases,^22,32,90,92−95^ and the presently gathered evidence joins previous reports that CD73-mediated formation of extracellular adenosine is a critical pathway to ensure the overactivation of A_2A_R^22−25^ together with the upregulation of A_2A_R.^24,32,90,92−95^

In conclusion, the present findings give further support to the contention that increased ATP release and ATP-derived formation of extracellular adenosine bolstering A_2A_R activation are a key pathway involved in abnormal synaptic plasticity in circuits that underlie behavioral dysfunction in different brain diseases, namely, upon repeated stress. Accordingly, new strategies to decrease abnormal ATP release, to inhibit CD73, or to antagonize A_2A_R are putative candidates to manage brain diseases. Future work should attempt providing an explanation for the mechanisms driving this concluded upregulation of the pathway of ATP release/CD73 activity/A_2A_R activation under stressful conditions. One possible reason may be related to a maladaptive attempt to preserve synapse stability under stressful conditions associated with atrophy and dysfunction of glutamatergic synapses,^47^ given that increased ATP release and CD73 and A_2A_R upregulation and overfunction are critical controllers of synaptic stability during development^77^ and upon circuit remodeling in the adult.^74^

## Materials and Methods

### Animals

Male Wistar rats (adults, 220–250 g, *n* = 56) were obtained from Charles River (Barcelona, Spain) and were maintained in groups of 2–3 in the same cage at 23–25 °C, with 12 h light/12 h dark cycle and standard chow and tap water *ad libitum*. All procedures in this study were conducted in accordance with the principles and procedures outlined as “3Rs” in the guidelines of the European Union (2010/63/EU), FELASA, and ARRIVE and were approved by the Portuguese Ethical Committee (DGAV) and by the Institution’s Ethics’ Committee (ORBEA 138-2016/15072016). Since the behavioral alterations caused by the utilized protocol of restraint stress were so far only validated in male rats, the “3Rs” guidelines imposed the use of only male rats to obtain the first proof-of-concept supporting a role for CD73 in the control of behavioral alterations caused by repeated stress.

### Restraint Stress

The stress model consisted of a repeated physical restraint of rats, as done previously.^78^ The rats were individually placed in a room adjacent to their colony and immobilized in a 25 cm × 7 cm plastic bottle, with a plastic taper on the outside and a 1 cm hole at one end for breathing. After the termination of each daily restraint stress session, the rats were returned to their home cages. The schedule of subchronic restraint stress consisted of a daily 4 h immobilization period (between 10 a.m. and 4 p.m.) during 14 consecutive days, the time previously defined to be required to cause stable behavioral modifications for at least 1 week in adult male rats.^78^ Control age-matched rats were handled as their tested littermates except that they were not immobilized.

### Preparation of Synaptosomes

Hippocampal synaptosomes (purified synapses) were prepared as previously described.^96^ After deep anesthesia under halothane atmosphere, each rat was decapitated, the two hippocampi were dissected and homogenized in sucrose (0.32 M) solution containing 1 mM EDTA, 10 mM HEPES, 1 mg/mL bovine serum albumin (Sigma), pH 7.4 at 4 °C, supplemented with a protease inhibitor, phenylmethylsulfonyl fluoride (PMSF 0.1 mM), a cocktail of inhibitors of proteases (CLAP 1%, Sigma), and the antioxidant dithiothreitol (1 μM). The homogenate was centrifuged at 3000*g* for 10 min at 4 °C, and the resulting supernatant was further centrifuged at 14 000*g* for 12 min at 4 °C. The resulting pellet (P2 fraction) was resuspended in 1 mL of a 45% (v/v) Percoll solution in Krebs-HEPES buffer (140 mM NaCl, 5 mM KCl, 25 mM HEPES, 1 mM EDTA, 10 mM glucose; pH 7.4). After centrifugation at 14 000*g* for 2 min at 4 °C, the white top layer was collected (synaptosomal fraction), resuspended in 1 mL of Krebs-HEPES buffer, and further centrifuged at 14 000*g* for 2 min at 4 °C. The pellet was then resuspended in Krebs-HEPES solution. The purity of this synaptic fraction has been previously quantified as >95%.^96^

### ATP Release

The release of ATP was measured online using the luciferin-luciferase assay, as previously described.^22,46^ Briefly, a suspension containing synaptosomes, an ATP assay mix (with luciferin and luciferase; from Sigma), and Krebs-HEPES solution was equilibrated at 25 °C during 10 min to ensure the functional recovery of nerve terminals. The suspension was then transferred to a white 96-well plate, and measurements were performed in a luminometer (Victor3). After 60 s to measure basal ATP outflow, the evoked release of ATP was triggered with 32 mM KCl (isomolar substitution of NaCl in the Krebs-HEPES solution), a well-established neurochemical strategy to trigger optimal signal-to-noise calcium-dependent vesicular release from synaptosomes without damage to these artificial synaptic structures.^22,46^ The evoked release of ATP was calculated by integration of the area of the peak upon subtraction of the estimated basal ATP outflow.^22,46^

### Western Blot

Western blot analyses of hippocampal synaptosomes were performed as described previously.^22,46,97^ Briefly, the levels of ecto-5′-nucleotidase (CD73) were assessed using a previously validated rabbit polyclonal anti-CD73 antibody (1:300, Sigma, SAB5701396),^22^ and the levels of vesicular nucleotide transporters (vNUT, SLC17A9, solute carrier family 17 member 9) were estimated using a rabbit polyclonal anti-VNUT antibody (1:500; Santa Cruz Biotechnology, sc-86312),^97^ validated by others,^98^ after labeling with a secondary goat anti-rabbit IgG antibody conjugated with alkaline phosphatase (1:20,000) (GE Healthcare, Chicago, USA). The membranes were then stripped for reprobing with GAPDH (rabbit polyclonal, 1:3000, Abcam) as a loading control. Immunoreactive bands were detected after incubation of membranes with ECF reagent (GE Healthcare), on a Bio-Rad Chemidoc imaging system.

### Intracerebroventricular Drug Administration

The continuous intracerebroventricular (icv) administration of the CD73 inhibitor α,β-methylene ADP (AOPCP, from Sigma) was carried out using implanted Alzet pumps, as previously described.^46^ Briefly, AOPCP (100 μM, Sigma) was administered (0.25 μL/h for 18–20 days) directly into the right lateral ventricle through osmotic minipumps (model 1004; Alzet Corporation), placed in a subcutaneous pocket in the back, slightly posterior to the scapulae, and connected via polyethelene tubing to an intracranial cannulae (Alzet Brain Infusion Kit II) targeting the left lateral ventricle (antero-posterior = 0.0 relative to bregma; lateral = 1.5 mm to the midline; depth = 4.5 mm down from the surface of the skull). Control animals received a similar icv administration of saline.

### Behavior

We used a tight schedule of behavioral characterization, with the minimal time interval between each test that avoided cross-interference between the tests.^32,37^ Restraint stress began 3 days after the placement of the Alzet pumps and was carried out from 9 a.m. until 6 p.m. during 14 consecutive days. Behavioral tests were carried out on the 15th until the 18th days after beginning the restraint stress protocol. Tests were carried out by two experimenters who were unaware of the phenotypes or drug treatments, in a sound-attenuated room with an 8 lx illumination and visual cues on the walls, to which the animals were previously habituated. The apparatuses were cleaned with 20% ethyl alcohol to remove any odors after testing each animal.

Locomotion and exploratory behaviors were monitored on the morning of day 15 using an open-field arena made of dark gray PVC measuring 100 × 100 cm^2^ (divided by white lines into 25 squares of 20 × 20 cm^2^) that was surrounded by 40 cm high walls. Each rat was placed in the center of the open field, and the following variables were recorded for 10 min: number of peripheral squares (adjacent to the walls) crossed (peripheral locomotion), number of central squares (away from the walls) crossed (central locomotion), and total locomotion (peripheral locomotion plus central locomotion).

Hippocampal-dependent memory was evaluated using the object displacement test, carried out in the afternoon of day 15. Rats were exposed to two identical objects in the same open field apparatus in which they were habituated and were allowed to explore for 5 min the objects fixed in opposite corners 20 cm away from walls and 50 cm apart from each other. In the test trial, carried out 2 h later, rats were again placed for 5 min in the open field arena except that one of the objects was moved to a novel position. Memory performance was quantified with an object displacement index defined as the ratio between the time exploring the object in the novel location over the total time exploring both objects. Exploration of an object is defined as directing the nose to the object at a distance equal to or less than 2 cm from the object and/or touching it with the nose; rearing onto object was not considered exploratory behavior.

Anxiety was further assessed in the morning of day 16, using the elevated-plus maze, where each animal was allowed to explore the maze for 8 min. The number of entries and the time spent in both open and closed arms were recorded, considering an entry only when the whole body and four paws were inside an arm.

Anhedonic-like behavior was evaluated in the afternoon of day 16 using the sucrose preference test, where rats were first single housed into a cage with two bottles. After 4 h of habituation, one bottle was randomly switched to contain 1.2% sucrose solution and the total consumption of water and sucrose solution was measured at the end of a 16 h test period (12 h dark phase plus 4 h light phase). Sucrose preference was calculated as the ratio of sucrose vs total intake.

The depressive-like behavior was evaluated in the forced swimming test, carried out in the morning of day 17. Rats were placed in individual glass cylinders (40 cm in height and 17 cm in diameter) containing water (water depth was 30 cm, kept at 25 ± 1 °C) to measure the total duration of immobility, climbing and swimming during a 10 min session. A rat was regarded as immobile when floating motionless or making only those movements necessary to keep its head above the water. The climbing behavior was defined as upward-directed movements of the forepaws along the side of the swimming chamber, and the swimming behavior was defined as movement (usually horizontal) throughout the swimming chamber; diving and face shaking behaviors were not considered.

Rats were sacrificed in scrambled pairs (1 control, 1 AOPCP-treated, 1 stressed, 1 stressed and AOPCP-treated) between days 18 and 20 after the start of treatment, by decapitation after deep halothane anesthesia.

### Electrophysiology

Rats were decapitated after anesthesia, and the brain was quickly placed in ice-cold, oxygenated (95% O_2_, 5% CO_2_) artificial cerebrospinal fluid (ACSF, in mM: 124.0 NaCl, 4.4 KCl, 1.0 Na_2_HPO_4_, 25.0 NaHCO_3_, 2.0 CaCl_2_, 1.0 MgCl_2_, 10.0 glucose). Transversal dorsal hippocampal slices (400 μm thick) were obtained using a McIlwain tissue chopper (Brinkmann Instruments, NY, USA), and coronal slices containing the prelimbic medial prefrontal cortex (PFC, 300 μm thick) were cut using a Leica VT1200S vibratome and placed in an holding chamber with oxygenated ACSF. Slices were allowed to recover at 32–34 °C for at least 1 h prior to recording, when they were transferred to a submerged recording chamber and superfused at 3 mL/min with oxygenated ACSF kept at 30.8 °C.

The configuration of the extracellular recordings was as previously described: in the hippocampus, the stimulating bipolar concentric electrode was placed in the proximal CA1 *stratum radiatum* for stimulation of the Schaffer collateral fibers, and the recording electrode, filled with 4 M NaCl (2–5 MΩ resistance), was placed in the CA1 *stratum radiatum* targeting the distal dendrites of pyramidal neurons;^99^ in the prefrontal cortex, the stimulation electrode was placed in layer II/III and the recording electrode was placed in layer V.^100^ Stimulation was performed using either a Grass S44 or a Grass S48 square pulse stimulator (Grass Technologies) or a Digitimer DS3 stimulator (Digitimer LTD), with rectangular pulses of 0.1 ms applied every 15–20 s. After amplification (ISO-80, World Precision Instruments, U.K., or AxoPatch 200B amplifier, Axon Instruments, USA), the recordings were digitized (PCI-6221 acquisition board, National Instruments or Digidata 1322A, Axon Instruments), averaged in groups of 3–4, and analyzed using either with the ClampFit version 10.5 program (Axon Instruments) or the WinLTP version 2.10 software.^101^ The intensity of stimulation was chosen between 40 and 50% of maximal field excitatory postsynaptic potential (fEPSP; in the hippocampus) or population spike (PS) response (in the prefrontal cortex), determined based on input/output curves in which the percentage of maximum fEPSP slope or PS amplitude was plotted versus stimulus intensity.

Long-term potentiation (LTP) was induced by high-frequency stimulation (100 Hz for 1 s) in hippocampal synapses;^102^ in the PFC, LTP was induced by delivering a train of 100 Hz (50 pulses, 0.5 s duration) for a priming effect, which was 15 min later followed by four trains of 100 Hz (50 pulses, 0.5 s duration, 1 every 10 s).^103^ LTP magnitude was quantified as the percentage change between two values: the average slope or amplitude of the 10 averaged potentials taken after LTP induction (between 50 and 60 min in the hippocampus and between 35 and 45 min in the prefrontal cortex) in relation to the average slope of the fEPSP or the PS amplitude measured during the 10 min that preceded LTP induction. The effect of drugs on LTP was assessed by comparing LTP magnitude in the absence and presence of the tested drugs in experiments carried out in different slices from the same animal. The drugs were tested at previously defined supramaximal but selective concentrations, namely, 100 μM AOPCP^4^ (Sigma), 50 nM SCH58261^104^ (Tocris), and/or 2 U/mL adenosine deaminase^105^ (Sigma).

### Statistics

Data are presented as the mean ± SEM of *n* experiments (i.e., *n* different rats). The comparison of ATP release from control and stressed rats and the effect of drugs was analyzed using a two-tailed unpaired Student’s *t* test with Welsh correction, whereas alterations of protein density were analyzed with a one-tailed *t* test compared to 100%. When testing the impact of AOPCP on the behavioral effects of stress, the data were first analyzed with a two-way ANOVA followed by a Newman–Keuls post hoc test. The statistical analysis of LTP magnitudes was carried out using a two-tailed *t* test to compare control and stressed rats and a paired *t* test to estimate the effects of either AOPCP or SCH58261 in stressed rats. All tests were performed using Prism 6.0 software (GraphPad, San Diego, CA, USA) considering significance at a 95% confidence interval.

## Notes

The data are available from the corresponding author upon reasonable request.
